# Supplementary material for: Predicting stress in first-year college students using sleep data from wearable devices
Source: PLOS Digit Health. 2024 Apr 11;3(4):e0000473. doi: 10.1371/journal.pdig.0000473 (PMC11008774; doi:10.1371/journal.pdig.0000473)
Supplement: S1 Text — (DOCX) [file pdig.0000473.s001.docx]

***Stress Assessment*** (modified Perceived Stress Score-10 to reduce recall period to 1 week [1].

Stress was assessed with the following questions. For each item, the participants selected (0 = Never, 1 = Almost Never, 2 = Sometimes, 3 = Fairly Often, 4 = Very Often). PSS scores are obtained by reversing responses (e.g., 0 = 4, 1 = 3, 2 = 2, 3 = 1 & 4 = 0) to the four positively stated items (items 4, 5, 7, & 8) and then summing across all scale items. A short 4 item scale can be made from questions 2, 4, 5 and 10 of the PSS 10 item scale.

1. In the last week how often have you been upset because of something that happened unexpectedly?
2. In the last week how often have you felt that you were unable to control the important things in your life?
3. In the last week how often have you felt nervous and "stressed"?
4. In the last week how often have you felt confident about your ability to handle your personal problems? (Reverse)
5. In the last week how often have you felt that things were going your way? (Reverse)
6. In the last week how often have you found that you could not cope with all the things that you had to do?
7. In the last week how often have you been able to control irritations in your life? (Reverse)
8. In the last week how often have you felt that you were on top of things? (Reverse)
9. In the last week, how often have you been angered because of things that were outside of your control?
10. In the last week how often have you felt difficulties were piling up so high that you could not overcome them?

1. Cohen S, Kamarck T, Mermelstein R. A Global Measure of Perceived Stress. J Health Soc Behav [Internet]. 1983 Dec [cited 2023 Mar 29];24(4):385. Available from: http://www.jstor.org/stable/2136404?origin=crossref
