# Supplementary material for: Predicting stress in first-year college students using sleep data from wearable devices
Source: PLOS Digit Health. 2024 Apr 11;3(4):e0000473. doi: 10.1371/journal.pdig.0000473 (PMC11008774; doi:10.1371/journal.pdig.0000473)
Supplement: S2 Text — (DOCX) [file pdig.0000473.s002.docx]

**Additional Methods**

***Nonlinear models*.** We also used GPBoost [1] to create mixed effects models that use gradient-boosted trees obtained using the LightGBM algorithm [2] rather than a linear model for the fixed effects, thus integrating nonlinearity into the models. We divide the number of users with 80% of them in the training set (*n*=405) and 20% in the test set *(n*=102). With the train and test sets having no common participants, we ensure that the model’s performance is not artificially inflated by learning patterns specific to a particular individual. While the number of weeks per participant varies, this split results in a roughly 80-20 split as well for the number of participant-week combinations in each set (*n_train_*=2074, *n_test_*=529).

The input data comprises various metrics for each user, for each week. These metrics include sleep measures from the Oura ring as well as information from the weekly survey and the baseline survey. In addition to requiring at least 3 days of sleep data per week, we also required at least 3 weeks of survey data per participant. The survey and the sleep data were then merged, and only participant-week combinations where there was both survey and sleep data were included in the analysis.

The data was grouped by a set of grouping variables, and we used a combination of measures as an input to a GPBoost binary classifier to predict the binary outcome of a PSS score of at least 14 (moderate or high stress). We considered three different sets of grouping variables: participant ID; participant ID and week number; and no grouping variable (equivalent to just using LightGBM). The Bernoulli probit likelihood was used for modeling the random effects. For modeling the fixed effects using LightGBM, we used 5-fold cross-validation to optimize the hyperparameters for the area under the receiver operating characteristic curve (ROC AUC), which is a measure of how well a binary classifier works across different discrimination thresholds. For a dummy classifier assigning every case to the dominant class, the area under the curve (AUC) is 0.5, even with class imbalance.

The split in the cross-validation was done on the unique user IDs to ensure that the training and validation sets did not have overlapping users. The LightGBM hyperparameters optimized are the learning rate (“learning_rate”={0.01, 0.1}), minimum samples in a leaf (“min_data_in_leaf”={10, 100, 1000}), and the maximum tree depth (“max_depth”={1, 2, 3, 5, 10}), allowing for a maximum of 2^10^ leaves (“num_leaves”); the optimum number of boosting iterations is then obtained by GPBoost’s grid_search_tune_parameters function, which uses an early stopping condition of 10 rounds in the cross-validation step. To assess whether the relationship between sleep measures and stress followed a nonlinear relationship, we used GPBoost [^34^](https://www.zotero.org/google-docs/?nXVYwN), a mixed effects model that uses gradient-boosted trees, to estimate the most dominant predictors for feature importance between all sleep measures and PSS. These models did not identify any sleep measures as dominant features in the prediction of PSS when compared to individual traits.

We used different sets of input variables for the GPBoost model. For input variables that were obtained from the Oura ring, we use all the summary statistics per participant as inputs. We also used other inputs that are constant for each participant as used in the linear models. The variables used for each model are given (S8 Table). Both the raw sleep durations and the percentage of total sleep hours were assessed for the sleep stages (i.e., REM, deep, light). Aside from using the raw sleep duration for REM and deep sleep, we also explored using the percentage of sleep assigned to these stages. The performance of each model was evaluated through the associated ROC AUC.

We fed all input features into GPBoost: sleep metrics, personality indicators, existence of prior mental health diagnosis, demographic factors (gender, ethnicity, race, first-generation status). For sleep metrics, we also consider using either the raw hours for the sleep stages or the percentage of total sleep spent in each sleep stage. For measuring feature importance, we used the total gains of splits which use a particular feature. The most dominant predictors are those whose feature importance measure is at least 50% of that of the predictor with the highest feature importance. In addition, we also ran the prediction using only sleep metrics. The models were evaluated using ROC AUC as the performance metric (S8 Table).

1. Sigrist F. Gaussian Process Boosting [Internet]. arXiv; 2022 [cited 2023 Apr 10]. Available from: http://arxiv.org/abs/2004.02653

2. Ke G, Meng Q, Finley T, Wang T, Chen W, Ma W, et al. LightGBM: A Highly Efficient Gradient Boosting Decision Tree. 2017;
