# Supplementary material for: Predicting stress in first-year college students using sleep data from wearable devices
Source: PLOS Digit Health. 2024 Apr 11;3(4):e0000473. doi: 10.1371/journal.pdig.0000473 (PMC11008774; doi:10.1371/journal.pdig.0000473)
Supplement: S1 Fig — (DOCX) [file pdig.0000473.s004.docx]

**Participants with survey and Oura data**. A) The first and second week were weeks of enrollment and baseline survey and therefore they had no overlapping participants. B) All participants were able complete up to 7 surveys.

**
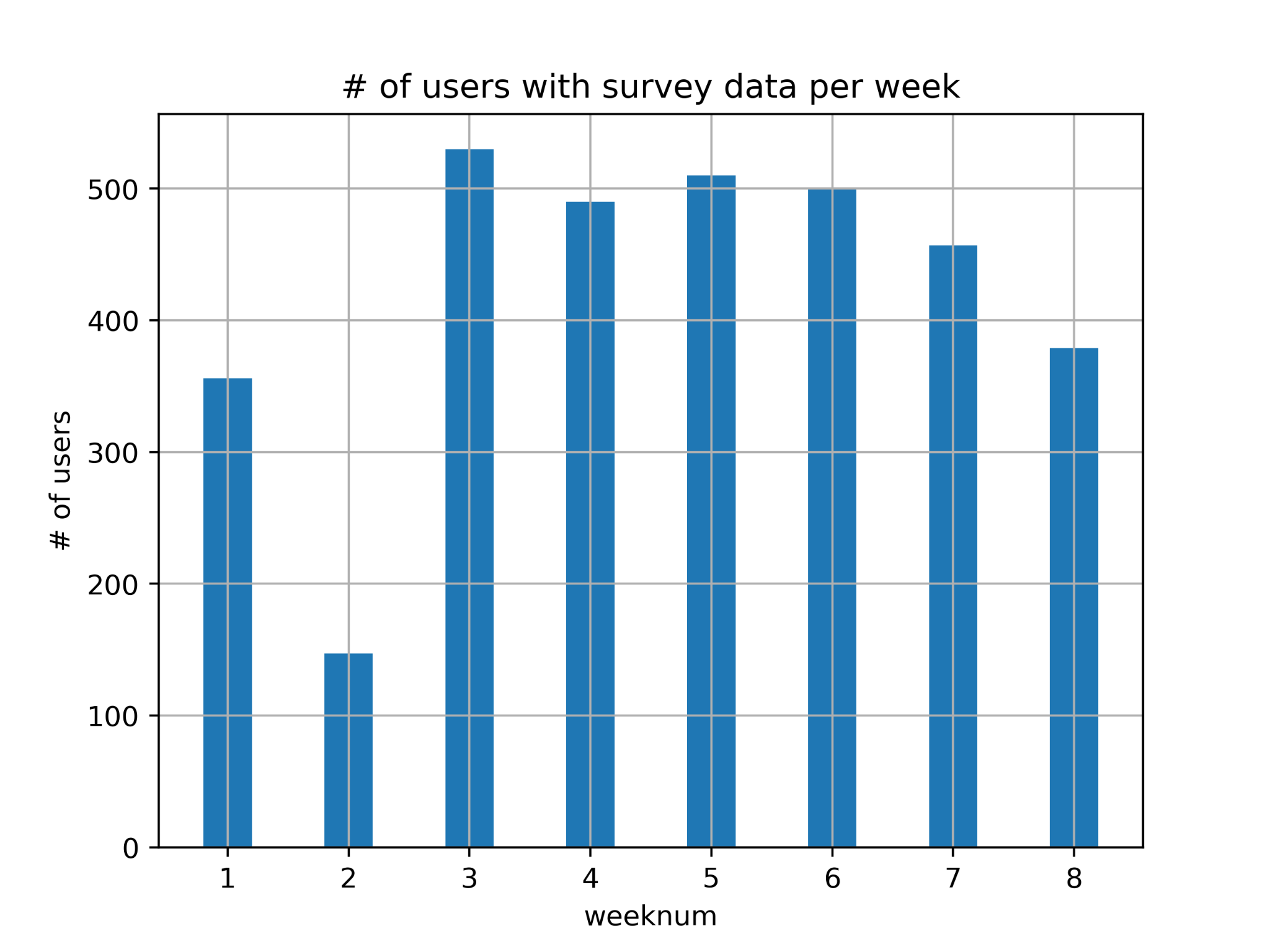

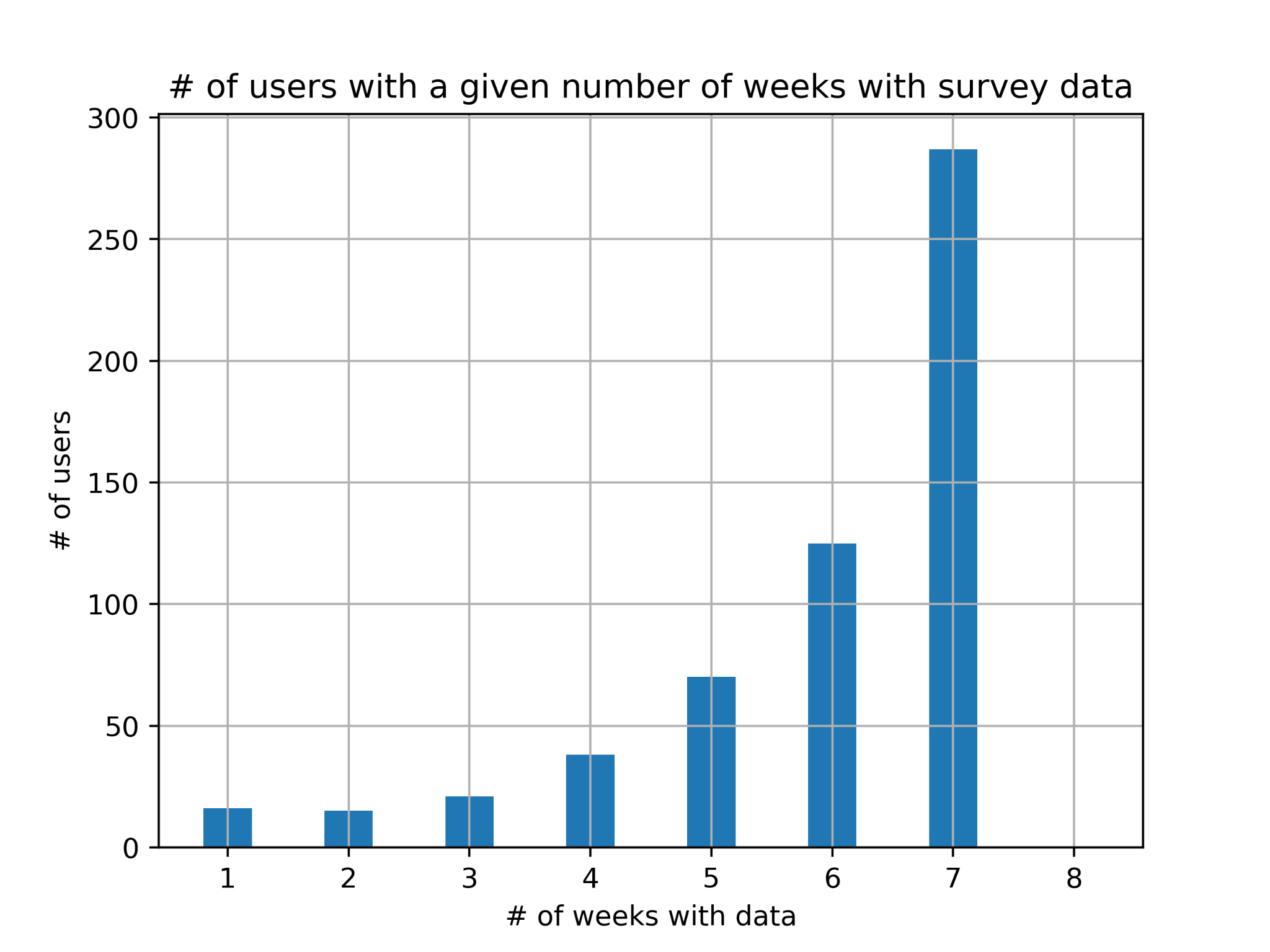
**

A

B
