# Supplementary material for: Predicting stress in first-year college students using sleep data from wearable devices
Source: PLOS Digit Health. 2024 Apr 11;3(4):e0000473. doi: 10.1371/journal.pdig.0000473 (PMC11008774; doi:10.1371/journal.pdig.0000473)
Supplement: S1 Table — (DOCX) [file pdig.0000473.s005.docx]

**Oura Ring measures, descriptions and units.**

| *Measure* | *Label* | *Description* | *Units* |
| --- | --- | --- | --- |
| Total sleep duration | total | Total amount of sleep registered during the sleep period, excluding periods when the participant is awake | seconds |
| REM sleep duration | rem | Total amount of REM sleep registered during the sleep period. | seconds |
| Deep sleep duration | deep | Total amount of deep (N3) sleep registered during the sleep period. | seconds |
| Bedtime start time | bedtime_start_delta | Duration before (-) or after (+) midnight when the sleep period started | seconds |
| Bedtime end time | bedtime_end_delta | Duration before (-) or after (+) midnight when the sleep period ended | seconds |
| Sleep onset latency | onset_latency | Detected latency from the detected start of bedtime to the beginning of the first five minutes of persistent sleep. | seconds |
| Sleep efficiency | efficiency | The percentage of the sleep period in sleep | percent |
| Number of times waking up | wake_up_count | The number of times the user woke up from bed during the sleep period. | count |
| Number of times getting up | got_up_count | The number of times the user got up during the sleep period. | count |
| Restlessness | restless | Percentage of sleep time when the user was moving, where a higher value is indicative of increased movement during sleep. | percent |
| Lowest heart rate | hr_lowest | Lowest heart rate recorded during the sleep period | Beats per minute |
| Average heart rate | hr_average | Average heart rate recorded during the sleep period | Beats per minute |
| Average heart rate variability | rmssd | Average heart rate variability recorded during the sleep period using the RMSSD method | milliseconds |
| Average respiratory rate | breath_average | Average respiratory rate | Breaths per minute |
| Average breathing rate variation | average_breath_variation | Average breathing rate variation | Breaths per minute |
| Skin temperature deviation (long-term) | temperature_delta | Skin temperature deviation from long-term average temperature | Celsius |
| Skin temperature deviation (3-day) | temperature_trend_deviation | Skin temperature deviation from 3-day rolling average temperature | Celsius |
