# Supplementary material for: Predicting stress in first-year college students using sleep data from wearable devices
Source: PLOS Digit Health. 2024 Apr 11;3(4):e0000473. doi: 10.1371/journal.pdig.0000473 (PMC11008774; doi:10.1371/journal.pdig.0000473)
Supplement: S2 Table — (DOCX) [file pdig.0000473.s006.docx]

**Distributions of outcome variables and sleep measures by dataset inclusion and exclusion criteria for participants with at least 3 nights of Oura data per week.**

| Inclusion Criteria: 3+ weeks Survey Responses, 3+ nights of Oura data per week | | | | | | | | |
| --- | --- | --- | --- | --- | --- | --- | --- | --- |
|  | Participants = 525  Oura data = 3,112 | | Participants = 511  Oura data = 2,959  Exclusion: No Weekends | | Participants = 507  Oura data = 2,603 Exclusion: No Thanksgiving Week | | Participants = 493  Oura data = 2,469  Exclusion: No Weekends or Thanksgiving Week | |
| Variable | **Mean** | **SD** | **Mean** | **SD** | **Mean** | **SD** | **Mean** | **SD** |
| PSS | 15.939 | 7.358 | 15.851 | 7.333 | 16.317 | 7.306 | 16.233 | 7.264 |
| PSS >=14 | 0.641 | 0.480 | 0.636 | 0.481 | 0.661 | 0.473 | 0.657 | 0.475 |
| Δ PSS | -0.480 | 5.900 | -0.512 | 5.864 | -0.407 | 5.497 | -0.264 | 5.243 |
| σ PSS | 0.000 | 4.115 | 0.000 | 4.070 | 0.000 | 3.851 | 0.000 | 3.814 |
| **Raw Estimates** |  |  |  |  |  |  |  |  |
| Total Sleep (Hrs) | 7.406 | 0.827 | 7.438 | .858 | 7.331 | 0.785 | 7.358 | 0.811 |
| Average HR | 63.143 | 8.519 | 62.746 | 8.643 | 63.078 | 8.565 | 62.664 | 8.694 |
| HRV | 66.968 | 31.953 | 68.244 | 32.826 | 67.098 | 32.145 | 68.479 | 33.073 |
| ARR | 15.583 | 1.586 | 15.556 | 1.607 | 15.582 | 1.596 | 15.559 | 1.617 |
| **Deviation in Estimates** |  |  |  |  |  |  |  |  |
| Total Sleep (Hrs) | 0.013 | 0.542 | 0.040 | 0.598 | -0.003 | 0.463 | 0.019 | 0.514 |
| Average HR | 0.055 | 3.030 | -0.321 | 3.303 | 0.035 | 2.920 | -0.348 | 3.235 |
| HRV | -0.177 | 9.800 | 0.890 | 10.801 | -0.181 | 9.570 | 0.909 | 10.727 |
| ARR | 15.583 | 1.586 | 15.556 | 1.607 | 15.582 | 1.596 | 15.559 | 1.617 |
| **Variance in Estimates** |  |  |  |  |  |  |  |  |
| Total Sleep (Hrs) | 3.297 | 1.476 | 2.723 | 1.399 | 3.277 | 1.474 | 2.675 | 1.375 |
| Average HR | 10.154 | 6.449 | 7.895 | 5.616 | 10.025 | 6.340 | 7.733 | 5.448 |
| HRV | 33.152 | 23.708 | 26.839 | 20.642 | 32.934 | 23.732 | 26.506 | 20.451 |
| ARR | 1.251 | 0.746 | 1.020 | 0.651 | 1.240 | 0.723 | 1.004 | 0.625 |
