# Supplementary material for: Predicting stress in first-year college students using sleep data from wearable devices
Source: PLOS Digit Health. 2024 Apr 11;3(4):e0000473. doi: 10.1371/journal.pdig.0000473 (PMC11008774; doi:10.1371/journal.pdig.0000473)
Supplement: S3 Table — (DOCX) [file pdig.0000473.s007.docx]

**Distributions of outcome variables and sleep measures by dataset inclusion and exclusion criteria for participants with at least 1 night of Oura data per week.**

| Inclusion Criteria: 3+ weeks Survey Responses, 1+ night Oura data | | | | | | | | |
| --- | --- | --- | --- | --- | --- | --- | --- | --- |
|  | Participants = 557  Oura data = 3,161 | | Participants = 556  Oura data = 3,032  Exclusion: No Weekends | | Participants = 557  Oura data = 2,688  Exclusion: No Thanksgiving Week | | Participants = 556  Oura data = 2,572  Exclusion: No Weekends or Thanksgiving Week | |
| Variable | **Mean** | **SD** | **Mean** | **SD** | **Mean** | **SD** | **Mean** | **SD** |
| PSS | 15.939 | 7.358 | 15.900 | 7.345 | 16.344 | 7.308 | 16.291 | 7.288 |
| PSS >=14 | 0.641 | 0.480 | 0.638 | 0.481 | 0.660 | 0.474 | 0.657 | 0.475 |
| Δ PSS | -0.480 | 5.900 | -0.523 | 5.859 | -0.258 | 5.257 | -0.272 | 5.253 |
| σ PSS | 0.000 | 4.115 | 0.000 | 4.058 | 0.000 | 3.832 | 0.000 | 3.785 |
| **Raw Estimates** |  |  |  |  |  |  |  |  |
| Total Sleep (Hrs) | 7.406 | 0.827 | 7.431 | 0.863 | 7.320 | 0.793 | 7.346 | 0.819 |
| Average HR | 63.143 | 8.519 | 62.766 | 8.627 | 63.121 | 8.535 | 62.711 | 8.658 |
| HRV | 66.968 | 31.953 | 68.121 | 32.814 | 67.006 | 32.127 | 68.213 | 32.97 |
| ARR | 15.583 | 1.586 | 15.564 | 1.601 | 15.586 | 1.590 | 15.566 | 1.608 |
| **Deviation in Estimates** | |  |  |  |  |  |  |  |
| Total Sleep (Hrs) | 0.013 | 0.542 | 0.040 | 0.599 | -0.004 | 0.467 | 0.019 | 0.518 |
| Average HR | 0.055 | 3.030 | -0.339 | 3.316 | 0.031 | 2.932 | -0.366 | 3.249 |
| HRV | -0.177 | 9.800 | 0.941 | 10.794 | -0.149 | 9.593 | 0.967 | 10.701 |
| ARR | 15.583 | 1.586 | 15.564 | 1.601 | 15.586 | 1.590 | 15.566 | 1.608 |
| **Variance in Estimates** | |  |  |  |  |  |  |  |
| Total Sleep (Hrs) | 3.297 | 1.476 | 2.721 | 1.396 | 3.274 | 1.477 | 2.682 | 1.379 |
| Average HR | 10.154 | 6.449 | 7.944 | 5.65 | 10.130 | 6.408 | 7.823 | 5.522 |
| HRV | 33.152 | 23.708 | 26.869 | 20.662 | 33.150 | 23.836 | 26.603 | 20.588 |
| ARR | 1.251 | 0.746 | 1.024 | 0.653 | 1.250 | 0.728 | 1.015 | 0.630 |
