# Supplementary material for: Predicting stress in first-year college students using sleep data from wearable devices
Source: PLOS Digit Health. 2024 Apr 11;3(4):e0000473. doi: 10.1371/journal.pdig.0000473 (PMC11008774; doi:10.1371/journal.pdig.0000473)
Supplement: S4 Table — (DOCX) [file pdig.0000473.s008.docx]

**Correlation between predictor variables and PSS.**

| Variables | (1) | (2) | (3) | (4) | (5) | (6) | (7) |  |
| --- | --- | --- | --- | --- | --- | --- | --- | --- |
| (1) PSS | 1.000 |  |  |  |  |  |  |  |
| (2) Gender (non-male) | -0.112 | 1.000 |  |  |  |  |  |  |
| (3) Week | -0.094 | -0.018 | 1.000 |  |  |  |  |  |
| (4) TST (hours) | -0.050 | -0.199 | 0.058 | 1.000 |  |  |  |  |
| (5) HR average (bpm) | 0.124 | -0.297 | 0.044 | 0.199 | 1.000 |  |  |  |
| (6) HRV (ms) | -0.099 | 0.109 | -0.030 | -0.170 | -0.705 | 1.000 |  |  |
| (7) ARR (breaths per minute) | 0.080 | -0.120 | -0.021 | 0.064 | 0.232 | -0.142 | 1.000 |  |
| Spearman rho = -0.142 | | | | | | | | |
