# Supplementary material for: Predicting stress in first-year college students using sleep data from wearable devices
Source: PLOS Digit Health. 2024 Apr 11;3(4):e0000473. doi: 10.1371/journal.pdig.0000473 (PMC11008774; doi:10.1371/journal.pdig.0000473)
Supplement: S5 Table — Model Yij = β0 + β1 X sleep measureij + β2 X weeknumij + u j + εij. (DOCX) [file pdig.0000473.s009.docx]

**Mixed-effects MLR models with single sleep measure (N=3,112) with 525 participants. Model** *Y_ij_* = *β*_0_ + *β_1_* X sleep measure*_ij_* + *β*_2_ X weeknum*_ij_* + *u_j_* + *ε_ij_*

| Predictor | Coef. | St.Err. | z | p-value | [95% Conf | Interval] |
| --- | --- | --- | --- | --- | --- | --- |
| Total sleep time (hrs) | -0.766 | 0.141 | -5.44 | **0.000** | -1.042 | -0.490 |
| Bedtime Start Time (min past midnight) | 0.259 | 0.107 | 2.42 | **0.015** | 0.050 | 0.468 |
| Bedtime End Time (min past midnight) | -0.229 | 0.100 | -2.30 | **0.022** | -0.425 | -0.034 |
| Wake up count | -0.199 | 0.058 | -3.41 | **0.001** | -0.313 | -0.085 |
| Average HR (bpm) | 0.080 | 0.021 | 3.81 | **0.000** | 0.039 | 0.121 |
| HRV (ms) | -0.016 | 0.006 | -2.60 | **0.009** | -0.028 | -0.004 |
| ARR (breaths/min) | 0.369 | 0.134 | 2.77 | **0.006** | 0.108 | 0.631 |
| Skin temperate deviation (degrees C) | 1.288 | 0.451 | 2.86 | **0.004** | 0.404 | 2.172 |
