# Supplementary material for: Predicting stress in first-year college students using sleep data from wearable devices
Source: PLOS Digit Health. 2024 Apr 11;3(4):e0000473. doi: 10.1371/journal.pdig.0000473 (PMC11008774; doi:10.1371/journal.pdig.0000473)
Supplement: S6 Table — (DOCX) [file pdig.0000473.s010.docx]

**Demographic variables as predictors of PSS.** Gender included as a binary variable is a significant predictor of PSS.

| PSS | Coef. | | St.Err. | t-value | | p-value | [95% Conf | | Interval] | | Sig |
| --- | --- | --- | --- | --- | --- | --- | --- | --- | --- | --- | --- |
| Gender (non-male) | 3.18 | | 0.295 | 10.77 | | 0.000 | 2.601 | | 3.760 | | *** |
| Race (non-white) | 0.071 | | 0.116 | 0.62 | | 0.537 | -0.156 | | 0.299 | |  |
| First generation college | -0.328 | | 0.451 | -0.73 | | 0.467 | -1.212 | | 0.556 | |  |
| Constant | 13.6 | | 0.256 | 53.12 | | 0.000 | 13.098 | | 14.102 | | *** |
|  | | | | | | | | | | | |
| Mean dependent var | | 15.939 | | | SD dependent var | | | 7.358 | |  |  |
| R-squared | | 0.036 | | | Number of obs | | | 3112 | |  |  |
| F-test | | 38.876 | | | Prob > F | | | 0.000 | |  |  |
| Akaike crit. (AIC) | | 21145.291 | | | Bayesian crit. (BIC) | | | 21169.463 | |  |  |
| **** p<.01, ** p<.05, * p<.1* | | | | | | | | | | | |
