# Supplementary material for: Predicting stress in first-year college students using sleep data from wearable devices
Source: PLOS Digit Health. 2024 Apr 11;3(4):e0000473. doi: 10.1371/journal.pdig.0000473 (PMC11008774; doi:10.1371/journal.pdig.0000473)
Supplement: S7 Table — (DOCX) [file pdig.0000473.s011.docx]

**Proportion of variance in outcome and predictor variables attributable to differences between subjects.** Intraclass Correlation Coefficients (ICC), Standard Errors, and 95% Confidence Intervals for variables to describe the proportion of total variability attributable to between subject differences.

|  |  |  | **PSS** | | **PSS-Mod** | |
| --- | --- | --- | --- | --- | --- | --- |
| Predictor | ICC (SE) | 95% CI | ICC (SE) | 95% CI | ICC (SE) | 95% CI |
| **Including Weekend (N=3112, 525 participants)** | | | 0.626 (0.018) | [0.591, 0.659] | 0.676 (0.026) | [0.624, 0.724] |
| **Raw Values** |  |  |  |  |  |  |
| Total Sleep (Hrs) | 0.505 (0.020) | 0.467, 0.544 | 0.631 (0.017) | 0.596, 0.664 | 0.685 (0.025) | [0.634, 0.733] |
| Average HR | 0.853 (0.009) | 0.835, 0.869 | 0.620 (0.018) | 0.585, 0.654 | 0.669 (0.026) | [0.617, 0.719] |
| HRV | 0.891 (0.007) | 0.877, 0.904 | 0.623 (0.018) | 0.588, 0.657 | 0.673 (0.026) | [0.620, 0.721] |
| ARR | 0.930 (0.004) | 0.921, 0.938 | 0.623 (0.018) | 0.588, 0.657 | 0.673 (0.026) | [0.620, 0.722] |
| **Variance** |  |  |  |  |  |  |
| Total Sleep (Hrs) | 0.236 (0.019) | 0.201, 0.275 | 0.626 (0.018) | 0.591, 0.660 | 0.626 (0.018) | [0.591, 0.660] |
| Average HR | 0.264 (0.019) | 0.227, 0.304 | 0.626 (0.018) | 0.591, 0.660 | 0.626 (0.018) | [0.591, 0.660] |
| HRV | 0.541 (0.019) | 0.503, 0.578 | 0.627 (0.018) | 0.591, 0.659 | 0.626 (0.018) | [0.591, 0.659] |
| ARR | 0.206 (0.018) | 0.171, 0.245 | 0.627 (0.018) | 0.591, 0.660 | 0.627 (0.018) | [0.592, 0.660] |
| **Excluding Weekend (N=2,959, 511 participants)** | | | PSS | | PSS-Mod | |
| **Raw Values** | ICC (SE) | 95% CI | ICC (SE) | 95% CI | ICC (SE) | 95% CI |
| Total Sleep (Hrs) | 0.468 (0.021) | 0.427, 0.509 | 0.633 (0.018) | 0.597, 0.667 | 0.683 (0.026) | [0.630, 0.731] |
| Average HR | 0.834 (0.010) | 0.814, 0.853 | 0.624 (0.018) | 0.589, 0.659 | 0.669 (0.026) | [0.615, 0.718] |
| HRV | 0.877 (0.008) | 0.861, 0.891 | 0.627 (0.018) | 0.591, 0.662 | 0.671 (0.026) | [0.617, 0.720] |
| ARR | 0.922 (0.005) | 0.911, 0.931 | 0.627 (0.018) | 0.591, 0.661 | 0.671 (0.026) | [0.617, 0.720] |
| **Variance** |  |  |  |  |  |  |
| Total Sleep (Hrs) | 0.229 (0.019) | 0.193, 0.269 | 0.629 (0.018) | 0.594, 0.663 | 0.626 (0.018) | [0.591, 0.660] |
| Average HR | 0.171 (0.018) | 0.138, 0.210 | 0.629 (0.018) | 0.594, 0.663 | 0.626 (0.018) | [0.591, 0.660] |
| HRV | 0.473 (0.021) | 0.433, 0.514 | 0.629 (0.018) | 0.594, 0.664 | 0.626 (0.018) | [0.591, 0.659] |
| ARR | 0.157 (0.018) | 0.124, 0.196 | 0.630 (0.018) | 0.595, 0.664 | 0.627 (0.018) | [0.592, 0.660] |
