# Supplementary material for: Predicting stress in first-year college students using sleep data from wearable devices
Source: PLOS Digit Health. 2024 Apr 11;3(4):e0000473. doi: 10.1371/journal.pdig.0000473 (PMC11008774; doi:10.1371/journal.pdig.0000473)
Supplement: S8 Table — (DOCX) [file pdig.0000473.s012.docx]

**Nonlinear predictive models in GPBoost for PSS outcome.**

| Model | Grouping | Features | Dominant Predictors | ROC AUC |
| --- | --- | --- | --- | --- |
| 1a | Participant ID, week number | Sleep measures (sleep duration in hours), mental health diagnosis, demographics | - Mental health diagnosis - Median bedtime start (deviation from user median) | 0.64 |
| 1b | Participant ID, week number | Sleep measures (sleep duration in hours) | - Median lowest heart rate - Median bedtime start (deviation from user median) - 5%ile temperature deviation - 5%ile RMSSD (deviation from user median) | 0.57 |
| 1c | Participant ID, week number | Sleep measures (sleep duration for sleep stage in % of total sleep), mental health diagnosis, demographics | - Mental health diagnosis | 0.63 |
| 1d | Participant ID, week number | Sleep measures (sleep duration for sleep stage in % of total sleep) | - Max REM duration - 95%ile average respiratory rate variation (deviation from user median) - Median bedtime start time (deviation from user median) - Max REM duration as % of total sleep (deviation from user median) - Median RMSSD (deviation from user median) | 0.55 |
| 2a | Participant ID | Sleep measures (sleep duration in hours), mental health diagnosis, demographics | - Mental health diagnosis | 0.62 |
| 2b | Participant ID | Sleep measures (sleep duration in hours) | - Median lowest heart rate - 5%ile RMSSD (deviation from user median) - Median RMSSD (deviation from user median) - 95%ile average respiratory rate variation (deviation from user median) - 95%ile bedtime start (deviation from user median) | 0.51 |
| 2c | Participant ID | Sleep measures (sleep duration for sleep stage in % of total sleep), mental health diagnosis, demographics | - Mental health diagnosis | 0.60 |
| 2d | Participant ID | Sleep measures (sleep duration for sleep stage in % of total sleep) | - Min average respiratory rate - Median lowest heart rate - Max REM duration as % of total sleep - Max REM duration as % of sleep (deviation from user median) - Min temperature trend deviation (deviation from user median) | 0.48 |
