# Supplementary material for: Predicting stress in first-year college students using sleep data from wearable devices
Source: PLOS Digit Health. 2024 Apr 11;3(4):e0000473. doi: 10.1371/journal.pdig.0000473 (PMC11008774; doi:10.1371/journal.pdig.0000473)
Supplement: Attachment — Submitted filename: Bloomfield_ResponsetoReviewers.pdf [file pdig.0000473.s013.pdf]

Raquel Simões de Almeida, PhD
Academic Editor
PLOS Digital Health

Dear Dr. Simões de Almeida,

Thank you for your thorough review of our original manuscript and for sharing the comments from the three reviewers. We have made several major revisions to the manuscript in order to meet the PLOS Digital Health Criteria and to respond to the concerns raised by the reviewers.

In order to meet the PLOS Digital Health Criteria, we have updated the Financial Disclosure statement, author sources of funding and role of funders in research, inclusion of data for reproduction of analyses, and required changes to document formatting.

In response to editor and reviewer comments, we have responded to each point below. One important note is that we removed a number of the psychological history predictors because they appeared to be confounders and were clouding the interpretation of results. We also used mean values of sleep estimates as opposed to the median values which changed the significance of many. And lastly, we changed the model structure. We hope that these many changes and responses to reviews has improved the manuscript and its potential impact substantially. We look forward to your review of these changes and greatly appreciate the time and energy you have spent reviewing our work.

Kind regards,

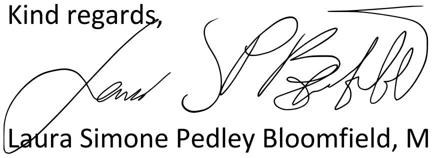

Laura Simone Pedley Bloomfield, MD/PhD
Assistant Professor of Research
University of Vermont

Response to Reviewers

<u>Editor Comments</u>

Journal Requirements

1. Please amend your detailed Financial Disclosure statement. This is published with the article. It must therefore be completed in full sentences and contain the exact wording you wish to be published.
    a. I have updated the Financial Disclosure statement to be in full complete sentences. It can be published in the current format.
2. State the initials, alongside each funding source, of each author to receive each grant.
    a. The funding source for the research and all authors funded by the associated grant has been updated. The official PIs on the grant that funded this study were Chris M. Danforth and myself, Laura S.P. Bloomfield.
3. State what role the funders took in the study. If the funders had no role in your study, please state: "The funders had no role in study design, data collection and analysis, decision to publish, or preparation of the manuscript."
    a. I have updated the statement to indicate that the funders had no role in the study design, data collection and analysis, decision to publish, or preparation of the manuscript.
4. If any authors received a salary from any of your funders, please state which authors and which funders .If you did not receive any funding for this study, please simply state: "The authors received no specific funding for this work."
    a. I have updated the funding statement to clarify that four of the authors have full or partial salary from the funders through the grant supporting the research.
5. We ask that a manuscript source file is provided at Revision. Please upload your manuscript file as a .doc, .docx, .rtf or .tex.
    a. I have uploaded the manuscript as a docx file.
6. Please provide separate figure files in .tif or .eps format only and remove any figures embedded in your manuscript file. Please also ensure that all files are under our size limit of 10MB.
    a. I have provided separate figure files in tif format and have removed all files that were embedded in the manuscript file.
7. We have noticed that you have uploaded Supporting Information files, but you have not included a list of legends. Please add a full list of legends for your Supporting Information files after the references list.
    a. We have now included a list of legends at the end of the manuscript file following the references.
8. In the online submission form, you indicated that "This is an ongoing study and therefore not all data will be available until the study conclusion to protect enrolled participants. Requests for data sharing and requests accessibility should be directed to the corresponding author". All

PLOS journals now require all data underlying the findings described in their manuscript to be freely available to other researchers, either 1. In a public repository, 2. Within the manuscript itself, or 3. Uploaded as supplementary information.

   a. We have provided data as supplementary information and with which results are replicable. We have removed all data that could breach compliance with our ethics board to maintain confidentiality of participants. Please let me know if this is not sufficient.

9. Firstly, they should address the unexpected association between perceived stress and autonomic markers, particularly the use of ARR instead of HR/HRV, providing explanations and considering potential measurement errors.
   a. Thank you for raising this critical point. It prompted us to delve deeper into the data and simplify our models in order to ensure that these associations were correct. We found that the removal of Mental Health History improved model fit.
10. Secondly, the authors should explore within-subject effects (if it is possible) to investigate how fluctuations in stress ratings over time affect autonomic markers, ensuring that the study capitalizes on its design for a more comprehensive analysis.
    a. For continuous measures of PSS, I have now included weekly fluctuations in PSS as two additional outcome measures: 1) Δ PSS and 2) σ PSS. Δ PSS is defined as the change in PSS from the previous measure and σ PSS is defined as the deviation of the measured value from the participant's mean.
11. Additionally, compliance and data collection details should be presented more prominently to establish the reliability of wearable device data.
    a. We observed a high level of compliance in this study. We filtered out participants who had less than 3 surveys in order to maintain integrity of the repeated measures analysis. 92.4% of participants completed at least three surveys (557/603). From the raw Oura data, 94.5% of user-week combinations (3530 out of 3735) had 3 or more Oura data points per week which corresponds to 98.7% (595) or the 603 participants with any days of Oura data. When weekly surveys were matched to weeks of Oura data with at least three nights of sleep data, 94.25% (525/557) of participants with at least three surveys were included. We have included these details in the current manuscript.
12. Furthermore, it's essential to differentiate between weekend and weekday sleep, given the distinctive sleep patterns among college students.
    a. There was no significant difference in the sleep measures when weekend days were included in the analysis. 96.31% of median values for each Oura measure are weekday measures. When creating mean weekly values for participants with three weekly Oura values, 93.32% of the data had 1 of the 3 days included as a weekend. We also completed the same analysis with data excluding weekends and there were no significant differences in the sleep measures included in the final regressions. For example, the full sample had a mean TST of 7.4 hours (SD=0.83). When weekends were removed the mean TST was 7.4 hours (SD=0.86). When Thanksgiving was removed the mean TST was 7.3 (SD=0.79). When both Thanksgiving and weekends were removed the mean TST was 7.4 (SD=0.81). Though, there may be significant differences in sleep

measures on the weekend and during the Thanksgiving break, removal of weekends and Thanksgiving from computing the mean values per week per participant did not significantly change our results, but did reduce the sample size by 7.6% (493/525).

13. Effect size, as well as statistical significance, should be calculated and presented to convey practical significance, addressing the wide variance in ARR relative to PSS. Other points made by the reviewers should be addressed. By attending to these key points, the paper will be better positioned to contribute significantly to the field.
    a. We assess the intraclass correlation coefficient (ICC) to assess the proportion of variance in the outcome variable PSS attributable to differences between subjects in the mixed-effects model. The proportion of total variability in the outcome variable PSS that is attributable to differences between subjects is ~63%. When weekends were included in the dataset: ICC = 0.626, SE = 0.017, and 95% CI [0.591, 0.659]. When weekends were excluded from the dataset: ICC = 0.629, SE = 0.018, and 95% CI [0.594, 0.663], Therefore, a substantial portion of variation in the outcome measure is due to differences between subjects and individual differences are important to PSS. Given the low SE, these are precise estimates. Since the ICC is significantly different from 0, there is substantial between-subject variability.
    b. However, many of the Oura measures have even higher ICC values (see Supplemental Information Table SX) indicating that the vast majority of variation is between subjects. The only validated sleep measure for which there is a substantial portion of the variation attributable to within-subject variability is total sleep time (TST) which has an ICC = 0.468 implying that 53.2% of its variation is within participants.
    c. When TST is added as a predictor to the base model of PSS, the ICC increases suggests that it explains some of the within-participant variability, but not a substantial amount, however, the predominant variability in PSS remains differences between participants.

<u>Reviewer #1 Comments</u>

14. A revealing study, current and relevant to the field of mental health and sleep studies, using a recent artificial intelligence device. However, there are still doubts about the prior validation by an ethics committee, as the opinion or registration number of the process submitted for evaluation was not provided in the annex, and, on the other hand, how the anonymity of the participants was guaranteed (procedures for storing, processing and accessing health/personal data were not specified).
    a. I have updated the methods and supplemental information with further information about anonymity of participants and security of data from our Data Safety Management Plan which was approved by the UVM IRB.
    b. We used REDCap to consent forms as well as baseline, weekly, and end-of-semester surveys. RedCAP is a HIPPA-compliant online application at University of Vermont. Each participant was assigned a unique study ID. The consent form is stored in a separate, password- protected folder. All data is stored on a secure virtual machine on a drive that has restricted access to those who are approved by the UVM IRB. Responses to

questions from electronic questionnaires are stored in REDCap. In both cases, data are identified with numerical IDs

c. When worn, the Oura ring automatically collects data of body responses during sleep and daily activity. That data is uploaded to Oura Cloud via the Oura mobile app and can be accessed using the Oura mobile app or Oura on the web. At enrollment in Oura Teams, all identifying information was removed and each participant was manually coded with their unique Participant ID given the potentially sensitive health information such as resting heart rate, sleep, and activity data. Oura Teams data is only accessed by those approved by the UVM IRB. Oura uses administrative, organizational, technical, and physical safeguards to protect the personal data that is collected and processed. Measures include encryption, pseudonymization and access right systems. Oura's security controls are designed to maintain an appropriate level of data confidentiality, integrity, availability, resilience and ability to restore the data. Oura regularly tests its service, systems, and other assets for security vulnerabilities.

15. On the other hand, nowhere in the article does it clarify the clinical validation of the OURA ring and its comparability with the PSG (the study identified as a reference for this validation was carried out on a small sample, in a different age group, especially in terms of behavior).
    a. We have included further
16. The study did not include exclusion criteria, which are mentioned here as one of the limitations. Instead of presenting this limitation, it should have been considered as a separate sub-group, or effectively eliminated in order to see if there were any changes to the results obtained. Even in terms of psychiatric pathology should be considered as an exclusion factor or analyzed as an independent group, given the incidence of some pathologies in the adolescence-adulthood transition.
    a. Thank you for this thoughtful comment. If I am understanding it correctly, There is concern that the previous mental health diagnosis should have served as an exclusion criteria. We did not exclude individuals from the study if they had a previous mental health diagnosis given that >40% of eligible participants (as this survey measure was taken during the baseline survey following enrollment) reported a history of mental health diagnosis, 90% of those being anxiety and/or depression. The goal of the intervention study was to provide mental health support to young adults who are at increased risk of mental health conditions and therefore it was not considered exclusionary.
    b. We completed subgroup analyses of the relationship between stress and sleep measures for those with and without a previous diagnosis of a mental health condition and the relationships did not hold in these subgroups. Thus we removed this predictor and simplified our models.

Reviewer #2: The authors report a large-scale observational study using Oura rings to measure sleep and nocturnal physiology, and self-report measures of perceived stress and mental health history to investigate the association between the objective measures of sleep and physiology and the subjective psychological measures. The findings show that sex, mental health history and average respiration rate

(ARR) are significantly associated with perceived stress. First, the authors must be commended for conducting a study such as this. Collecting data at this scale and over an extended period is no mean feat. The report is concise and to the point; however, there are several issues that need to be addressed. From a conceptual viewpoint items #3,

Design and analysis

1. I thought the literature review on prior work on autonomic markers of stress was excellent, as were references comparing the sensitivity of HR and respiration measures to different experimental stress tests. Given the knowledge that stress through sympathetic activation increases both HR, decreases HRV and increases respiratory rate, It is of major concern that the hypothesized association between stress (PSS) and HR/HRV was not found, but instead, an association was with ARR alone. We don't know the MAE of ARR measurement with Oura (we do know the errors relative to PSG for sleep and HR/HRV with ECG). Published data that the authors cite show that the MAE from PPG derived Respiration Rate is 2-3 breaths/min which is within the noise levels of the effect documented here. There needs to be some explanation for this especially when the latter is inferred from the PPG signal yields HRV data as well.
    a. Thank you for this thoughtful insight which led us to reconsider our analytical method and to focus on the variance within each of the predictors.
    b. We also removed factors that may have been confounders (i.e., mental health history and trauma history) and have consistent results for HR, HRV, and ARR as would be expected.
2. The value of a mixed effects model is often revealed in within-subject effects, in this case, instances where higher tri-weekly stress ratings would affect autonomic markers. Identifying how greater self-ratings of stress relate to differences in autonomic markers of stress would surely be interesting. The authors have the design to investigate fluctuations of stress over time. If this is what the authors are already showing, they should be explicit about it. If not, perhaps they could consider adding an analysis to test whether changes in PSS affect markers of interest.
    a. For continuous measures of PSS, I have now included weekly fluctuations in PSS as two additional outcome measures: 1) Δ PSS and 2) σ PSS. Δ PSS is defined as the change in PSS from the previous measure and σ PSS is defined as the deviation of the measured value from the participant's study mean. Unfortunately, we do not have daily measures of stress given that we have nightly measures of sleep which would provide more insight into the connection between stress ratings and autonomic markers. We plan to include an EMA design in future work to try and investigate more precise temporal resolution.
3. In a longitudinal study like this, a clear indication of compliance / acceptance needs to be stated in the results and not buried in Pg. 19 of supplementary material. Overall, the wear rate was quite good. It would also be good to know at the outset how many weeks (7) data was collected for.
    a. Thank you for noting the need for greater clarity on compliance. We have included a table in the supplemental information. We collected Oura data for 8 weeks. Participants

filled out a baseline survey which included the PSS assessment and then were sent a follow-up weekly survey for another 6 weeks. Therefore, the maximum number of surveys filled out by any participant was 7. 92.37% (557/603) of participants who enrolled in the study, completed at least 3 surveys. 36.64% (233/603) of enrolled participants completed all 7 surveys.

4. Regarding periods of non-wear of the rings. Data was included (and averaged across the week) if at least 3 days of data was present. Our experience with missing data is that it might not be an issue given the overall compliance here. However, this may be an issue if the missingness of the data is not random (e.g., if a participant removes the ring when they are studying late and forgets to put it back on). More data showing the extent and distribution of the missing data, and possibly analysis to show it is not a concern would help the reader have more confidence in the data.
    a. 94.5% of user-week combinations (3530 out of 3735) had 3 or more Oura data points per week. We only looked at nightly Oura data that had a long sleep period meaning that these 3 or more points had readings for an entire night. These correspond to 98.7% (595) of the 603 that have any Oura data.
5. Given that the sleep durations are quite good for college students, it would be helpful to differentiate between weekend and weekday sleep, which we know differ in timing and duration in this population. Including this in the analysis may provide more insight into sleep patterns and how they relate to stress (given their long TST, it may be that these students are primarily wearing the rings at the weekends).
    a. We appreciate the note that sleep varies by day of week, especially whether a night is on a weekend or a weekday. The data is well balanced by day of week with 28.69% of sleep data representing a weekend night which is consistent with 2/7 = 28.57% as we expect from unbiased wear. We have recompiled the entire dataset excluding weekend days and have rerun the regressions with this updated dataset. The sleep measures and results for the regressions do not significantly change with the removal of weekend days. We have included descriptive analyses for average total sleep time (TST), bedtime, and wake time with data excluding and including weekend days in Table 1 and regressions for both datasets within the Supplemental Information.
6. With large datasets, it is important to convey the effect size in addition to statistical significance as a significant factor that has a tiny effect size may not be practically meaningful. This should be calculated and displayed in the tables. Of concern is the scatter plot in Fig 1 shows the wide variance in ARR relative to PSS.
    a. In order to assess the effect size, we compared variance components of the models with and without each predictor. As you pointed out, the average variance for ARR is wide compared to PSS. As now shown in Table 1, a participant throughout the course of the study is 2.42 breaths per minute (SD = 1.03) and over the course of a week an average variance of 1.02 (SD= 0.65) per participant. Even though ARR continues to be a significant predictor of PSS in our models (provided in Supplemental Information), your concern that this significant relationship is meaningful is valid given the lack of studies comparing ARR estimated from the Oura ring with PSG. Additionally, we computed ICC

for each sleep measure and found that >90% of the variation seen in ARR was attributable to differences between participants. We re-ran analyses for weekly ARR variance and weekly ARR deviation from participant study mean ARR which remained significant.

7. As with point #3, it would be helpful if the model(s) was specified in the main text. Table 1 is not interpretable by itself without that information.
    a. I was unsure if you meant Table 1 or Table 3 given that Table 1 is descriptive and not based on a model. However, both Table 1 and Table 3 have been updated.
8. It would be helpful to see a table of the correlations between the various independent variables, to get an idea of the structure of the data. Higher ARR is probably correlated with being female, so that might explain the relationship (while that should be accounted for in the model, but with high collinearity, there might still be some effect). Knowing how the independent variables relate to each other would give the reader some idea about this.
    a. We appreciate your asking for clarity on the data structure. I have included a correlation table in the supplemental information. There are no correlations above 0.20 for the independent variables included in our final model.
9. In Table 1, and throughout, should probably be referred to as Heart Rate Variability. Also, in the lower half of Table 1, there is no indication what the values represent. I assume it is PSS score, but this should be stated clearly.
    a. Table 1 has been revised. RMSSD there and throughout the manuscript has been changed to HRV. Also the main outcome variable has been labeled as PSS.
2. When comparing models, predictors are usually added to models, which are then tested against the simpler model to see if they add explanatory power. A slightly different approach is taken in this paper, which isn't necessarily wrong; however, we don't know whether adding mental health history to the demographic factors significantly improves the model fit.
    a. Thank you for giving this helpful feedback. Your comments have provided an impetus for even greater analysis and we are grateful for greater depth of understanding for the relationships we have explored. We had included stepwise tables for the addition of each variable to the regression.
3. The first sentence of the abstract is not correct. Recent works by Yap (Ann Behav Med 2022), Menghini (Sleep Health 2023), Ng (Sleep Medicine 2023) have sought to relate sleep to mental-health markers/ stress. Also, in the abstract, the language describing the relationship between ARR, and stress seems to suggest that higher ARR results in stressful experiences, when the relationship is probably the other way round.
    a. You are correct that stress is the hypothesized driver of changes in sleep measures. I have modified the language to reflect this hypothesized relationship. Given that I have re-expanded the focus of the paper to include TST, HR, and HRV this has also been updated.
4. Wearables do not measure contributors to sleep disturbances or mental health – all they do is provide estimates of sleep (and physiological) measures. Discovering what contributes to sleep disturbance and mental health issues requires significantly more.

    a. We really appreciate your attention to the clarity of language and have updated the language to reflect that we have used estimates of s

5. The title is similarly imprecise, the study does not aim to detect stress, rather it attempts to predict perceived stress. Furthermore, the data collected in this study is not wearable – it is collected using wearable devices.
    a. I appreciate your attention to language precision and believe these edits will improve the clarity of the paper. I have modified the title to reflect your recommendations.

Reviewer #3: this is well written paper in important topic

6. I am interested in the internal consistently and intraclass correlation of the data structure
    a. Thank you for this feedback. I have included a table of the intraclass correlation to better represent the data structure included in the Supplemental Information.
7. discussion need to be developed further
    a. I have revised the discussion to include our additional analyses, however, if there are particular areas that you see are missing or need to be further expanded upon, we would be happy to include.
8. references need attention for their format intext
    a. I have re-added and relinked the references in-text to ensure that the references are standardized. My apologies, transitions between formats have often unlinked references and I appreciate your understanding that these were not correctly formatted in our first submission. We hope this has improved, but we are amenable to making further changes as necessary.
