# Supplementary material for: Predicting stress in first-year college students using sleep data from wearable devices
Source: PLOS Digit Health. 2024 Apr 11;3(4):e0000473. doi: 10.1371/journal.pdig.0000473 (PMC11008774; doi:10.1371/journal.pdig.0000473)
Supplement: Attachment — Submitted filename: Bloomfield_ResponsetoReviewers_02-07-24.pdf [file pdig.0000473.s014.pdf]

Raquel Simões de Almeida, PhD
Academic Editor
PLOS Digital Health

Dear Dr. Simões de Almeida,

Thank you for your thorough review of our revised manuscript and for sharing the comments needed to make the manuscript acceptable for publication.

In response to editor and reviewer comments, we have responded to each point below. We have re-reviewed the references to ensure that they are all up to date and do not include any retracted articles. Secondly, we have modified the structure of the manuscript sections to comply with journal standards.

We hope that these changes and our responses to final comments has improved the manuscript and its potential impact. We look forward to your review of these changes and greatly appreciate the time and energy you have spent reviewing our work.

Kind regards,

Laura Simone Pedley Bloomfield, MD/PhD
Assistant Professor of Research
University of Vermont

Response to Reviewers

<u>Editor Comments</u>

Journal Requirements

1. Please review your reference list to ensure that it is complete and correct. If you have cited papers that have been retracted, please include the rationale for doing so in the manuscript text, or remove these references and replace them with relevant current references. Any changes to the reference list should be mentioned in the rebuttal letter that accompanies your revised manuscript. If you need to cite a retracted article, indicate the article's retracted status in the References list and also include a citation and full reference for the retraction notice.
    a. We have reviewed and redownloaded all the references and none that are cited in the final revised manuscript are shown to be retracted. However, please advise if we have any references that do not meet the journal's criteria for submission.

Additional Editor Comments (if provided):

2. I have had the opportunity to thoroughly review the revised manuscript titled "Predicting stress in college freshmen using sleep data from wearable devices" and I am pleased to report that the authors have diligently addressed all the comments provided by the reviewers, and the overall quality of the paper has significantly improved as a result. Nevertheless, the authors placed the "Materials and Methods" section after the "Conclusions", something that I don't think is very appropriate and that doesn't happen in other articles published on PLOS Digital Health. However, and despite this change which I think is necessary, I am confident in recommending its acceptance for publication. The manuscript now meets the high standards of the journal, and its dissemination would undoubtedly contribute valuable insights to the relevant academic community.
    a. Thank you for your careful read of the revised manuscript. We are grateful for all of the feedback which helped us to improve our paper and its ability to make meaningful contribution.
    b. We have revised the order of the sections and the "Materials and Methods" section is now before the "Conclusions."
